# Biliprotein maturation: the chromophore attachment

**DOI:** 10.1111/j.1365-2958.2008.06160.x

**Published:** 2008-04-01

**Authors:** H Scheer, K-H Zhao

**Affiliations:** 1 Department Biologie I, Universität München Menzinger Straße 67, D-80638 München, Germany; 2 College of Life Science and Technology, Huazhong University of Science and Technology Wuhan 430074, China

## Abstract

Biliproteins are a widespread group of brilliantly coloured photoreceptors characterized by linear tetrapyrrolic chromophores, bilins, which are covalently bound to the apoproteins via relatively stable thioether bonds. Covalent binding stabilizes the chromoproteins and is mandatory for phycobilisome assembly; and, it is also important in biliprotein applications such as fluorescence labelling. Covalent binding has, on the other hand, also considerably hindered biliprotein research because autocatalytic chromophore additions are rare, and information on enzymatic addition by lyases was limited to a single example, an EF-type lyase attaching phycocyanobilin to cysteine-α84 of C-phycocyanin. The discovery of new activities for the latter lyases, and of new types of lyases, have reinvigorated research activities in the subject. So far, work has mainly concentrated on cyanobacterial phycobiliproteins. Methodological advances in the process, however, as well as the finding of often large numbers of homologues, opens new possibilities for research on the subsequent assembly/disassembly of the phycobilisome in cyanobacteria and red algae, on the assembly and organization of the cryptophyte light-harvesting system, on applications in basic research such as protein folding, and on the use of phycobiliproteins for labelling.

## Introduction

Biliproteins are a widespread group of photoreceptors characterized by linear tetrapyrrolic chromophores, bilins, which are covalently bound to the apoproteins via thioether bonds. The phycobiliproteins are photosynthetic antenna pigments that allow cyanobacteria, red and cryptophyte algae to efficiently harvest light in the ‘green gap’ where chlorophylls absorb only poorly, thereby contributing a substantial fraction to global photosynthesis (MacColl and Guard-Friar, 1987; Sidler, 1994). Phytochromes, the other group of biliproteins, were originally characterized as red/far-red photosensory receptors in green plants, but have since been found to be much more widespread in algae, bacteria and even fungi (Lamparter, 2004; Rockwell *et al*., 2006). There is also an increasing number of phytochrome-related biliproteins that absorb in different regions of the visible spectrum (Rockwell *et al*., 2006; Ishizuka *et al*., 2007; Montgomery, 2007). Phycobiliproteins and phytochromes are functionally and phylogenetically only distantly related; however, they not only possess very similar chromophores, but also share the same mode of covalent binding to and similar interactions with their apoproteins. Unlike chlorophylls, free bilins, as well as denatured biliproteins, are photophysically unsuited as photoreceptors: they absorb light only poorly and their excited states are very short-lived, thereby leading to rapid conversion of excitation energy to heat. The photophysical properties of native biliproteins are, by contrast, much more favourable: the light absorption of the chromophores is increased by almost one order of magnitude and the excited lifetimes by four orders of magnitude which, in combination, render them excellent photoreceptors. The principles underlying these molecular adaptations are still only partly understood, but involve extensive chromophore protein interactions by which the chromophore conformation and dynamics are modulated.

Covalent binding to the apoproteins appears to be an important factor assisting these interactions. Mutants in which a cysteine-binding residue has been replaced, for example, by serine, or mutants lacking lyases indicate that covalent binding is not absolutely necessary for function (Gindt *et al*., 1994; Jorissen *et al*., 2002; Inomata *et al*., 2006; Shen *et al*., 2008). It is, however, part of the functional optimization, because it stabilizes both the labile chromophores (Scheer, 1982) and proteins (Anderson and Toole, 1998; Shen *et al*., 2008).

The relatively stable covalent thioether bond is also important for biliprotein applications. Many protein separation techniques have been tested with the brilliantly coloured and intensely fluorescing phycobiliproteins, and they are used for fluorescence labelling (Oi *et al*., 1982; Wilson *et al*., 1996; Tooley *et al*., 2001). The pronounced pigment–protein interactions in the native state and the presence of multiple, often differently absorbing chromophores, rendered these pigments attractive biophysical models. A covalent bond is advantageous, for example, when studying protein folding (Ma *et al*., 2007; Kupka and Scheer, 2008), because it prevents chromophore dissociation in the denatured state where non-covalent interactions are minimized. The covalent bond, on the other hand, has also considerably hindered advances in biliprotein research. Unlike the chlorophylls, retinal and fluorescent proteins, there were no general methods available, until recently, for directed chromophore modifications. In particular, methods were lacking for reconstituting the holo-phycobiliproteins from the component chromophore(s) and apoprotein(s).

In cyanobacteria and red algae, up to four bilin chromophores are post-translationally attached, via thioether bonds, to specific cysteines of as many as a dozen, or even more, individual proteins. The focus of this review is the covalent attachment of the free bilin chromophores, which is the major part of an extensive series of post-translational modifications that also include methylation of a conserved asparagine-72 in the β-subunits (Swanson and Glazer, 1990) and, in some allophycocyanin (APC) α-subunits, removal of the N-terminal methionine (Sidler, 1994; Shen *et al*., 2008).^1^ Research in this field has recently gained momentum by the discovery of new types of lyases for chromophore attachment. We summarize the current status, emerging concepts and questions, with a focus on cyanobacterial phycobiliproteins and their implications for the assembly and degradation of the phycobilisome (PBS). Ten years ago, very little was known of this crucial process in biliprotein maturation but, recently, progress has been remarkable in defining both autocatalytic and lyase-dependent chromophore attachments.

### Structures of phycobiliproteins

**Fig. 1:**
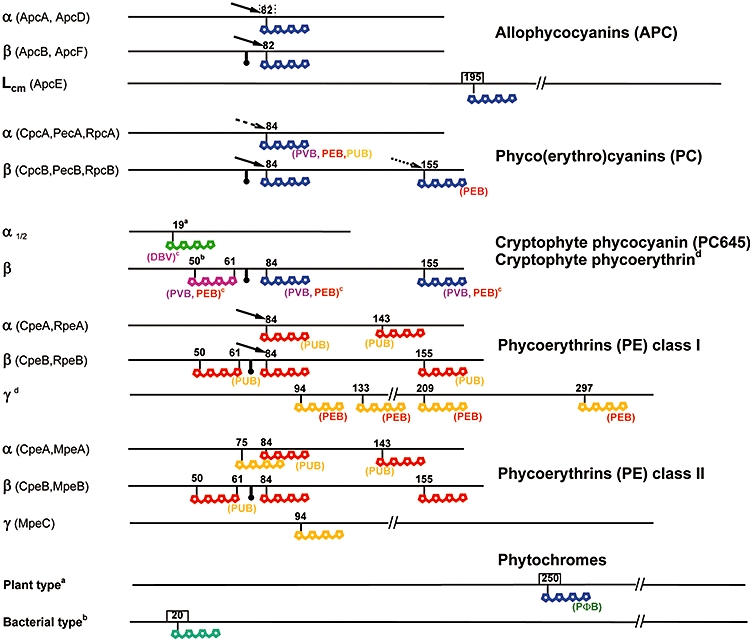
Post-translational modifications of biliproteins. Tetrapyrrole binding cysteines (consensus numbering^2^) with chromophores indicated in their approximate colours of the native chromoproteins (PCB in blue, PVB in purple, PEB in red, PUB in orange, MBV in green, BV in blue-green and PΦB in dark green): alternative chromophores to certain binding sites are indicated in brackets. Arrows pointing to tetrapyrrole binding sites represent identified lyases, with solid arrows indicating S-type lyases, dotted arrows T-type lyases and the dashed arrow E/F-type lyases. Boxed site numbers indicate correct autocatalytic binding, dotted boxes unclear situations. Vertical knobs indicate (partial) methylation at Asn-β72. Another PE, termed PE III, is not shown here. It has been identified in a high-light *Prochlorococcus marinus*, it carries only a single chromophore on the α-subunit, and none on the β-subunit (Hess *et al*., 1996). a, plant and most cyanobacterial phytochromes; b, bacterial, fungal and several cyanobacterial phytochromes; c, PE 545; d, only in red-algal b- (and possibly B-) PE. For biliprotein nomenclature, see Sidler (1994), Schluchter and Bryant (2002), and, alternatively (MacColl, 1998).

Phycobiliproteins from cyanobacteria and red algae are a large, monophyletic family of homologous heterodimeric proteins. Both the α- and β-subunits, which are also homologous to each other, consist of a globin-type core that carries the chromophore(s), and an N-terminal extension that is mainly involved in oligomerization. The subunits form heterodimers, and these can further oligomerize to ring-shaped ‘trimers’ (heterohexamers) and ‘hexamers’ (heterododecamers) that constitute the building blocks of the unique extra-membraneous antenna complex, the PBS (Scheer, 1982; Ficner and Huber, 1993; Sidler, 1994; Ritter *et al*., 1999; Stec *et al*., 1999; Wang *et al*., 2001; Adir *et al*., 2002; Nield *et al*., 2003; Doust *et al*., 2004; Schmidt *et al*., 2007). Oligomerization is largely reduced in the apoproteins; therefore, chromophore attachment also seems a prerequisite for PBS assembly (Anderson and Toole, 1998). The hexameric building blocks are further arranged in short stacks that form the PBS core, or to longer rods that are attached to the former. This supramolecular organization is mainly due to linker proteins which are located, as a central backbone, in the inner triangular hole of the ring-shaped biliproteins (Tandeau de Marsac and Cohen-Bazire, 1977; Sidler, 1994; Apt *et al*., 1995; Reuter *et al*., 1999; Liu *et al*., 2005). Most of the linker proteins are colourless, but at least two of them also carry covalently bound chromophores, namely, the core-membrane linker L_cm_ (= ApcE), and the γ-subunits of class II and some class I phycoerythrins (PEs) (Fig. 1). However, crystal structures of these phycobiliproteins have not been solved. Other, less characterized biliproteins, are variants of unknown function (Montgomery *et al*., 2004), or the PE of certain *Prochlorococcus* species, marine picocyanobacteria that lack PBSs (Hess *et al*., 2001). Cryptophyte phycobiliproteins represent a second type of biliprotein antenna with different structure and organization (Sidler, 1994): the β-subunits are phylogenetically related to the β-subunits of red algal PEs, but the α-subunits are much shorter and probably of different origin. The phytochromes and related sensory photoreceptors that form yet another group of phylogenetically unrelated biliproteins generally carry only a single chromophore at one of two alternative binding sites (Lamparter, 2004; Ishizuka *et al*., 2007; Montgomery, 2007).

**Fig. 2:**
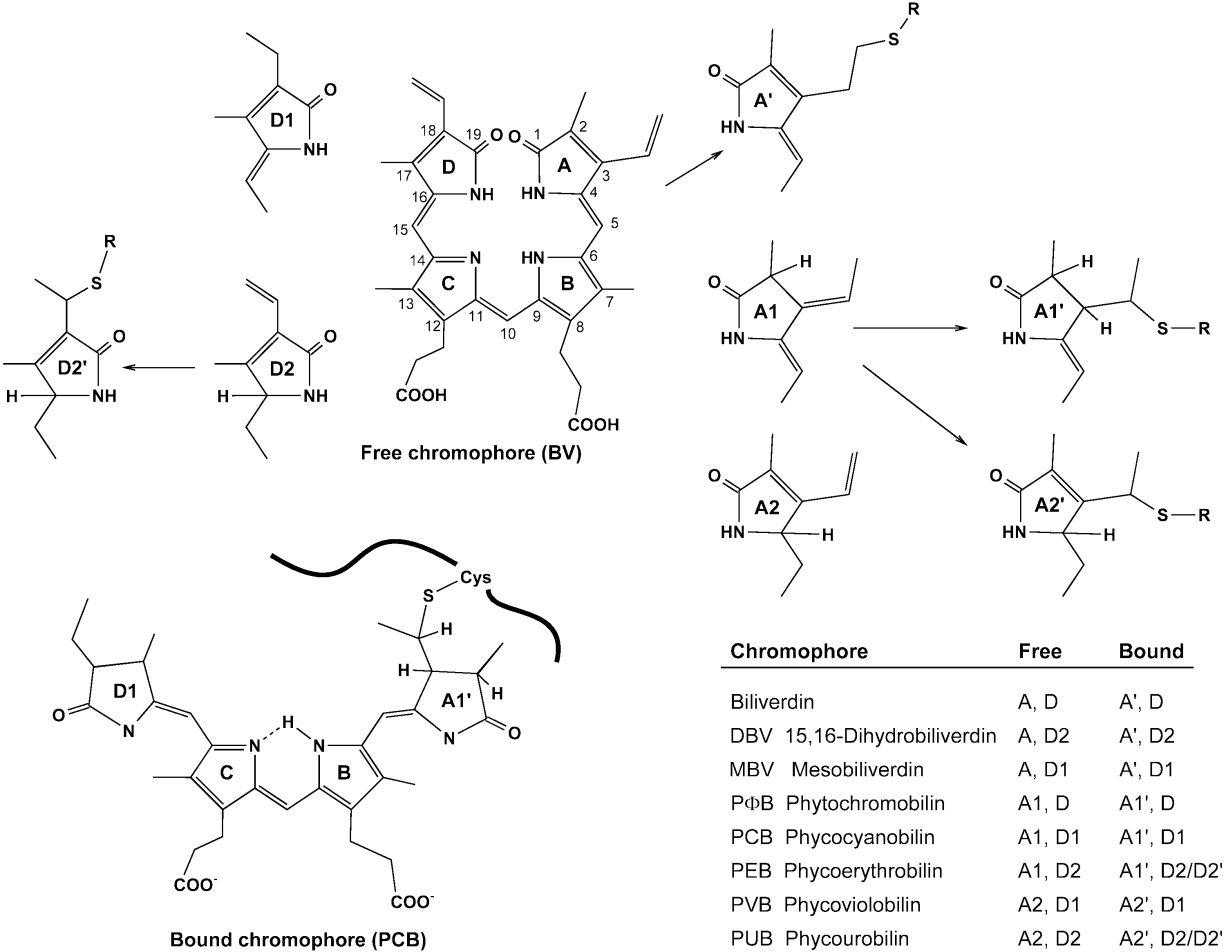
Free and protein bound bilins. Full structures of biliverdin in the typical cyclic-helical conformation of free chromophores (with IUPAC numbering, top centre), and of PCB bound at C-3^1^ to a cysteine residue of the apoprotein, in the typical extended conformation of bound chromophores (lower left), and partial structures of modified rings A and D in other biliprotein chromophores. Rings B and C remain unchanged throughout. Protein-linked rings are marked by ‘prime’, this nomenclature is also used in the lower right table giving the names and abbreviations of the various biliprotein chromophores in the free and protein-bound form. Arrows represent lyase actions; apoprotein is indicated by heavy wavy lines.

Individual phycobiliprotein subunits carry one to four chromophores (Fig. 1), with the number of binding sites increasing from APC to phycocyanin (PC) and phycoerythrocyanin (PEC) and further to PE, and cryptophyte biliproteins (Sidler, 1994). One conserved binding site, Cys-84,^2^ is present in all cyanobacterial and red algal phycobiliproteins, and is also present in the β-subunits of cryptophyte biliproteins. Additional binding sites have evolved in the globin domain by insertions near the C-terminus, around position 150, and towards the N-terminus, around position 50. Most chromophores are attached to the apoproteins' cysteine residues by a single thioether bond at C-3^1^ of the chromophore, but a second linkage is present in some PEs where a phycoerythrobilin (PEB, see Fig. 2 for chromophore structures and abbreviations), or phycourobilin (PUB) is bound at C-3^1^ to Cys-β50, and at C-18^1^ to Cys-β61 (Ficner and Huber, 1993). There are also exceptions where binding occurs to C-3^2^ of the C-3 side-chain; for example, biliverdin (BV) is bound via C-3^2^ in bacterial phytochromes (Lamparter *et al*., 2004; Wagner *et al*., 2005) and so is doubly bound 15,16-dihydrobiliverdin (DBV) in the cryptophyte biliproteins (Beale, 1993; Wemmer *et al*., 1993).

A single bond to C-3^1^ of the chromophore is also present in chromophorylated linker proteins and in (most) phytochromes. New asymmetric C-atoms with well-defined configurations are generated during the addition reaction to C-3^1^ (and C-18^1^). For example, the configuration of the newly generated chiral C-3^1^ is different in the two binding sites of β-CPC; namely, 3^1^(R) at the Cys-84 site, and 3^1^(S) at the Cys-155 site (Schirmer *et al*., 1987; Adir *et al*., 2002).

Binding is even more complex because homologous proteins can carry different chromophores at certain sites and, further, chromophore exchange at a particular site has been reported in response to a change in light quality (Everroad *et al*., 2006). In particular, in marine species, PUB (λ_max_ = 500 nm) can replace PEB (λ_max_∼540 nm), or PEB can replace phycocyanobilin (PCB) (λ_max_∼620 nm), to extend the absorption of the respective chromoproteins to shorter wavelengths, thereby adapting the organisms to the existing light environment. The largest variation is found at Cys-α84 of different cyanobacterial phyco(erythro)cyanins, to which four different chromophores, including the photoactive phycoviolobilin (PVB), can be bound.

In addition to this covalent binding, there are extensive non-covalent interactions between the apoprotein and the chromophore that are essential for biliprotein function. While free bilins adopt a rather flexible, cyclic-helical conformation (Falk, 1989), biliprotein chromophores assume rigid, extended conformations in the native biliproteins (Scheer, 1982; Ficner and Huber, 1993; Ritter *et al*., 1999; Stec *et al*., 1999; Wang *et al*., 2001; Adir *et al*., 2002; Nield *et al*., 2003; Doust *et al*., 2004; Wagner *et al*., 2005; Inomata *et al*., 2006; Schmidt *et al*., 2007). Variations in this basic conformation contribute to the fine-tuning of the absorption by which light harvesting or light perception is optimized. In the PBS, there exist both binding site-specific interactions and interactions with linker proteins (Reuter *et al*., 1999).

### Biosynthesis of phycobilins

Phycobilins are derived from protoheme (Beale, 1999; Frankenberg and Lagarias, 2003a). The macrocycle of protoheme is cleaved by a heme oxygenase that is homologous to the microsomal enzyme. In the recently discovered bacterial phytochromes with an N-terminal binding site, the resulting open chain tetrapyrrole, BV IXα, is bound without further modifications (Lamparter *et al*., 2004). In all other cases, BV is further reduced by ferredoxin-dependent bilin reductases at one or two out of three different positions (Fig. 2). Partial structure D1 is produced by reduction of the 18-vinyl group, partial structure D2 by reduction of the Δ15,16 double bond, and partial structure A1 by reductive isomerization of the Δ2,3/Δ3^1^,3^2^ diene system at ring A; the latter reaction yields the 3-ethylidene group characteristic of free PCB, PEB and phytochromobilin (PΦB). PCB is generated by a single reductase (PcyA) that catalyses two reductions, at ring A and the 18-vinyl group (Frankenberg and Lagarias, 2003b). PΦB reductase (Hy2) catalyses only reduction of ring A, leading to the typical chromophore of plant phytochromes (Kohchi *et al*., 2001), while a dedicated (yet unknown) reductase catalysing only reduction of the 18-vinyl group would yield mesobiliverdin (MBV), one of the chromophores of cryptophyte biliproteins. In PEB biosynthesis, two enzymens (PebA and PebB) generally act in sequence (Dammeyer and Frankenberg-Dinkel, 2006), but a bifunctional reductase catalyzing both steps has recently been identified in a Prochlorococcus cyanophage (Dammeyer *et al.*, 2008). The intermediate, DBV, is present in cryptophyte biliproteins, but the enzyme leading to its formation is unknown. PCB and PEB are the only free chromophores found in cyanobacteria and red algae but generally only in low amounts, and these occur in spite of two additional chromophores, PVB and PUB, being found in their biliproteins.

### Chromophore attachment

All chromophores are subsequently attached covalently to the apoproteins, but at least one of the missing chromophores (PVB), is generated during the attachment reaction by a simultaneous isomerization reaction (Zhao *et al*., 2002). Currently, there are four characterized modes of chromophore attachment.

#### 

##### Spontaneous attachment

It was recognized early that most apoproteins can bind phycobilins (PCB, PEB) spontaneously *in vitro*. This process, however, is of low fidelity and generally (see next section) leads to product mixtures (Arciero *et al*., 1988; Fairchild and Glazer, 1994a; Schluchter and Glazer, 1999). Side reactions include oxidations and incorrect stereochemistry at the asymmetric carbons that arise by the attachment. *In vivo*, spontaneous chromophore binding is unlikely because the chromophores are reactive, their concentrations are low, and biliprotein synthesis is a major metabolic pathway in cyanobacteria. Spontaneous attachment, however, constitutes a considerable problem in binding studies because it interferes, especially *in vitro*, with lyase assays and is not easily distinguished from truly autocatalytic lyase activities (Schluchter and Bryant, 2002; Böhm *et al*., 2007; Zhao *et al*., 2007a). As an example, autocatalytic binding has been reported for ApcA from two cyanobacteria, including *Nostoc* PCC7120 (Hu *et al*., 2006; Zhao *et al*., 2007). In our hands, however, the product differed distinctly in its absorption, fluorescence and circular dichroism from isolated α-APC, and correct attachment required the lyase, CpcS (see below).

Nonetheless, spontaneous binding provides important clues for understanding correct chromophore binding. First, it indicates that many, if not all, apoproteins can form thioether bonds with suitable chromophores. For certain applications like fluorescence labelling, the correct, native-like binding may not even be required if the lower fluorescence yield can be tolerated. Second, the bonds are formed with the correct cysteines, namely, only those located at the native binding sites, indicating site-specific interactions of the chromophores with the binding pockets. This is further emphasized by the non-covalent, yet (largely) functional binding of chromophores in mutants that lack the chromophore binding cysteine residue (Gindt *et al*., 1992; Lamparter and Michael, 2005; Shen *et al*., 2008), or the non-covalent binding of modified chromophores (Inomata *et al*., 2006). Obviously, these apoproteins can control the conformations of the chromophores and reduce their flexibility.

The importance of conformational control is emphasized by other lines of evidence. (i) The presence or absence of the detergent, Triton X-100, directs the chromophore preferentially to the Cys-155 or Cys-84 binding site, respectively, in the PC and PEC β-subunits (Zhao *et al*., 2004b). The conformation of the free chromophore (and, to a lesser extent, also that of the apoprotein) is changed by the detergent. It also inhibits the simple addition of PCB to PecA *in vitro*, thereby favouring its Isomerization, catalysed by the Cys-α84 lyase, PecE/F (Böhm *et al*., 2007) (see below). (ii) Some phytochromes bind the chromophore non-covalently in the dark, but an irradiation cycle induced covalent binding (Lamparter and Michael, 2005). During irradiation, there is probably not only the changed configuration of the Δ15,16 bond, but also a change in chromophore conformation. (iii) There is also evidence for non-covalent binding in biliproteins that slowly becomes covalent, conceivably by a conformational rearrangement in the binding pocket (Shen *et al*., 2008).

According to these data, an important, if not major, function of lyases (or lyase domains in phytochromes and ApcE) seems to be to guide the error-prone spontaneous attachment by conformational control of the chromophore, in a chaperone-like, but ATP-independent, fashion (Schluchter and Glazer, 1999). Direct evidence for such a chaperone-like function has been obtained recently with the E-subunit of an E/F-type lyase (Böhm *et al*., 2007). The situation is somewhat reminiscent of heme binding to apo-cytochrome *c* via two thioether bonds, which chemically resembles biliprotein binding in several respects. While a considerable number of proteins are involved in co-ordinating transport of apo-cytochromes and heme across the cytoplasmic membrane, and for ensuring their chemically precise covalent ligation (Turkarslan *et al*., 2006), they are not required for the actual heme binding *in vitro* under optimized conditions (Daltrop *et al*., 2002). With the c-cytochromes, redox control is considered a critical factor for the central Fe in the heme, and in avoiding formation of a disulfide bond within the heme binding motif Cys-Xaa-Xaa-Cys-His, whereas conformation seems the crucial factor in biliprotein maturation.

##### Autocatalytic attachment

Autocatalytic attachment is defined here as a spontaneous attachment leading to chromoproteins that are spectroscopically, biochemically and functionally indistinguishable from the respective native forms isolated from the parent organism (except for the lack of methylation of some β-subunits). Currently, correct chromophore binding, which is a true autocatalytic lyase activity, is mostly observed only among phytochromes (Wu and Lagarias, 2000), but it is the exception among phycobiliproteins (boxed sites in Fig. 1). In the phytochromes, two alternative binding sites are present. Interestingly, there is evidence that autocatalytic chromophore attachment in both types of phytochromes requires the co-action of domains that are near the respective alternative binding sites (Bhoo *et al*., 1997; Lamparter *et al*., 2004; Zhao *et al*., 2004a). In the crystal structure (Wagner *et al*., 2005), these domains are spatially close. Autocatalytic binding has also been found for ApcE, which could be reconstituted with PCB to give native-like L_CM_ (Zhao *et al*., 2005a). Both the chromophore carrying and the lyase domains reside in the N-terminal region of this large protein. Arguably, true autocatalytic binding may also occur with ApcA (Hu *et al*., 2006) (see *Spontaneous attachment*).

##### E/F-type lyases

In *Synechococcus* PCC 7002, a heterodimeric lyase (CpcE/F) is required for correct binding of PCB to Cys-α84 (Zhou *et al*., 1992). This was the first identified enzyme dedicated to chromophore attachment in biliproteins. The heterodimeric enzyme catalyses both the forward (binding) and the reverse (releasing) reaction (Fairchild *et al*., 1992). It also catalyses the addition of PEB to apo-α-CPC (CpcA), but with reduced affinity and kinetics compared with PCB (Fairchild and Glazer, 1994b). This E/F-type lyase has long served as a model structure in the search for lyases to other binding sites. As in *Synechococcus* PCC 7002 (Zhou *et al*., 1992), the genes, *cpcE* and *cpcF*, are located in several cyanobacteria, on the *cpc*-operon downstream from two genes (*cpcB/A*) coding for the apoproteins, and a third one (*cpcC*) coding for a linker protein (Kaneko *et al*., 1996; Hess *et al*., 1999; Kaneko *et al*., 2001). This is not true, however, for the *apc* and *cpe* operons, and often it is also not true for the *cpc* operons, especially in PE-producing strains. Homologous genes have also been found in other locations in several cyanobacteria (De Lorimier *et al*., 1993; Wilbanks and Glazer, 1993; Kahn *et al*., 1997), but their total numbers were always far too low to account for the number of different binding sites in the PBS (reviewed by Schluchter and Glazer, 1999).

The *pec* operons of *Nostoc* PCC7120 and *Mastigocladus laminosus* contain two homologous genes, *pecE/F* (Kufer *et al*., 1991), which code for a variant of this lyase (Zhao *et al*., 2002). PEC contains the photoactive PVB chromophore at Cys-α84, and PecE/F from these organisms not only attach PCB to this site but also simultaneously isomerize it to PVB. This probably explains why the free PVB chromophore had never been found in cyanobacteria. PUB is another example of such a chromophore: as the same Δ5-to-Δ2 double-bond isomerization that generates bound PVB from PCB would generate bound PUB from PEB, it is reasonable to speculate that this reaction is again catalysed by the action of an isomerizing lyase that uses PEB as substrate. Evidence for such a lyase has been found in marine cyanobacteria like *Synechococcus* sp. WH8102, which carries a fused *cpcEF* gene. The CpcF domain of the encoded protein has a motif that is characteristic for the isomerizing PecF subunit of the PVB:PecA lyase (Six *et al*., 2005). As these organisms lack PEC, another function could be the attachment and isomerization of PEB to generate protein-bound PUB, but biochemical evidence is still lacking. Two proteins homologous to CpcE and CpcF, namely CpeY and CpeZ, are encoded in the genomes of several PE-containing cyanobacteria and are implicated in PE chromophorylation (Wilbanks and Glazer, 1993; Kahn *et al*., 1997). However, these proteins have not been biochemically characterized.

##### Cyanobacterial S/U-type lyases

The high specificity and small number of E/F-type lyases in published genomes triggered the search for additional lyases in cyanobacteria. First results on new types of lyases in *Synechococcus* PCC7002 were presented only 4 years ago at a meeting (Shen *et al*., 2004). One of them, CpcS, is coded by a homologue of a gene, *cpeS* that, in *Fremyella diplosiphon*, is on an operon with a gene, *cpeR*, which had previously been associated with gene regulation (Cobley *et al*., 2002). Yet another *Synechococcus* gene is homologous to *cpeT* on the *Fremyella* operon; it codes for a third type of lyase (see below). The possible role in chromophore attachment of yet another protein, CpcV, which is encoded nearby is not yet resolved (Shen *et al*., 2008).

This information set the stage for the subsequent rapid characterization of lyases that now account, in principle, for chromophore attachment to all binding sites of APCs, CPCs, PECs, and even to some binding sites of CPEs (Shen *et al*., 2006; Zhao *et al*., 2006b; 2007a; Saunée *et al*., 2008; Shen *et al*., 2008).

The S/U type of lyases comprises a new family of proteins that are unrelated to the E/F-type lyases and exhibit rather large and characteristic variations (see below). Based on the nomenclature used by Cobley *et al*. (2002), they should be annotated as CpeS/U in PE-producing cyanobacteria, and CpcS/U in cyanobacteria lacking PE. The main feature of the S/U lyases is high binding site specificity, but a very low specificity for the chromophore and the receptor apoprotein. CpcS from *Nostoc* PCC 7120 is a nearly universal lyase for PCB attachment at Cys-84 of apo-phycobiliproteins (Zhao *et al*., 2007a). It correctly attaches PCB to almost all phycobiliproteins of the PBS core (ApcA1, ApcB, ApcD and ApcF), both in *Escherichia coli* and *in vitro*, and even a variant, ApcA2, which is induced under nitrogen starvation. The only exception is the large chromophorylated linker protein, L_CM_, which attaches the chromophore autocatalytically (see above). Furthermore, CpcS attaches PCB site-selectively to Cys-84 of CpcB and PecB, the β-subunits of CPC and PEC, and it can even attach PEB to the homologous site in CpeA and CpeB (Fig. 1). The broad selectivity of CpcS contrasts with the high specificity of the EF-type lyases for the cysteine-α84 site of CPC and PEC; it is matched by a unique motif of these biliprotein subunits near the binding site (Zhao *et al*., 2007a). Interestingly, the Y129C point mutation of CpcA rescues the phenotype of a mutant lacking CpcE, indicating that this site might now be served by a CpcS/U-type lyase (Swanson *et al*., 1992). The newly introduced cysteine is close to the C-3^1^ of the chromophore but deeply buried, the data did not support that it serves as an alternative binding site.

This broad substrate specificity has been supported by studies with CpcS from *Synechococcus* PCC 7002 (Saunée *et al*., 2008; Shen *et al*., 2008). Nonetheless, the two CpcS-type lyases studied so far seem to belong to two subtypes: CpcS from *Nostoc* PCC 7120 acts as a monomeric, single subunit lyase. Complex formation has been shown with other lyases from this organism but it always resulted in reduced activities (Zhao *et al*., 2006b). By contrast, CpcS from another organism, *Synechococcus* PCC 7002, is inactive on its own, and requires CpcU as a second subunit for activity. This functional heterogeneity is reflected by the phylogenetic classification of the two subtypes into different groups of the complex S/U protein family (see below) (Shen *et al*., 2008).

The finding of broad substrate specificity in two subclasses indicates that this might be a general property of the S-type lyases. Many organisms, in particular PE-containing ones, however, carry several homologous genes (see, e.g. Nakamura *et al*., 2003). The capacity of CpcS from the PE-less *Nostoc* PCC 7120 to attach PEB to both subunits of CPE might then only be an *in vitro* side reaction, while dedicated PE-lyases catalyse the reaction *in vivo*. It is noteworthy, however, that two reports exist on such homologues that do not seem to act as lyases (Zhao *et al*., 2007b; Saunée *et al*., 2008); the subject therefore requires further work. As one of the homologues is expressed under N-starvation, a function in biliprotein turnover is conceivable.

##### Cyanobacterial T-Type lyases

*Synechococcus* PCC7002 has yet another gene, *cpcT*, which is clustered with *cpcS* and *CpcU*. It is ubiquitous in cyanobacteria and codes for a third family of lyases, the T-type. Recombinant CpcT catalyses the regiospecific PCB addition at Cys-155 of CpcB from *Synechococcus* PCC7002 (Shen *et al*., 2006), and to Cys-155 of both β-CPC and β-PEC in *Nostoc* PCC 7120 (Zhao *et al*., 2007b). As CpcB and PecB are relatively close, these data indicate moderate protein specificity combined with a high site-specificity for the T-type lyases.

In PBS-containing cyanobacteria devoid of PE, three lyases seem to be sufficient to attach all phycobiliprotein chromophores: CpcS(/U) and CpcT attach PCB to Cys-β84 and Cys-β155, respectively, of PC and PEC, while CpcE/F and PecE/F catalyse attachment to Cys-α84 of PC and of PEC, respectively, the latter with a concomitant isomerization of PCB to PVB. The chromophore of L_cm_ is attached autocatalytically to the apoprotein, ApcE, and CpcS catalyses attachment to Cys84 of all the other biliproteins. A question immediately arises: what is the sequence of events. As chromophore-free apoproteins show severely reduced oligomerization (Toole *et al*., 1998), it is likely that chromophorylation precedes oligomerization. CpcB and PecB carry two chromophores, while PE and cryptophyte biliproteins up to four (Fig. 1). There is currently only a single experiment reported that addresses the question of binding order. For reconstitutions of both β-CPC and β-PEC, only a specific sequential attachment, first at Cys-β155 and then at Cys-β84, resulted in the correct product, and prior chromophorylation at Cys-β84 inhibited the subsequent attachment to Cys-β155 (Zhao *et al*., 2007b).

##### Lyases of other phycobiliprotein synthesizing organisms

Information on biliprotein lyases from red algae, picocyanobacteria and cryptophytes is still sparse. However, related genes have been found in members of each group (De Lorimier *et al*., 1993; Douglas *et al*., 2001; Kunimoto *et al*., 2003; Rocap *et al*., 2003; Copeland *et al*., 2005; Chisholm *et al*., 2006; Lane *et al*., 2008; Touchman, 2008), and even in a cyanobacteria-infecting virus (Mann *et al*., 2005). The respective genes are located in the plastid of rhodophytes and in the nucleomorph of cryptophytes. Also, homologous genes are found in photosynthetic organisms that do not contain phycobiliproteins, and even in non-photosynthetic organisms, but the sequence homologies are generally much poorer in these two latter cases than among genes of phycobiliprotein-containing organisms. This may indicate additional functions of the lyases. However, none of the respective gene products has to date been characterized functionally.

## Lyase mechanisms

Currently, there are very few mechanistic studies on biliprotein lyases published, and they have focused on CpcE/F from *Synechococcus* PCC 7002 (Fairchild and Glazer, 1994b; Schluchter and Glazer, 1999), on CpcE/F and PecE/F from *M. laminosus* and *Nostoc* PCC7120 (Böhm *et al*., 2007) and on CpcS1 from *Nostoc* PCC7120 (Tu *et al*., 2008) that has been classified as an S-type lyase of group III (Shen *et al*., 2008).

**Fig. 3:**
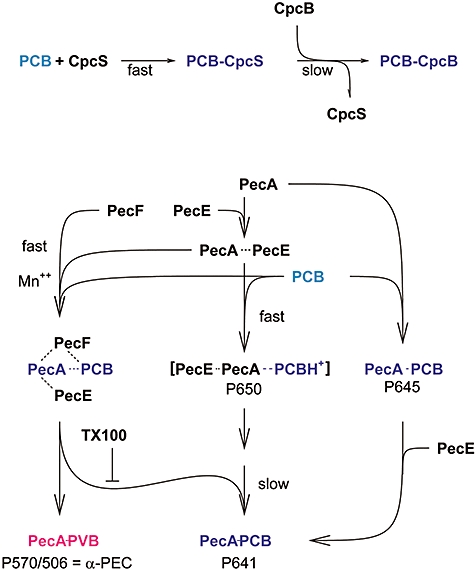
Reaction schemes of the S-type (top) and isomerizing E/F-type lyases (bottom). Non-covalent chromophore binding is indicated by broken lines, covalent binding by solid lines and colouration of both the chromophore and the protein. Intermediates (Pxxx) are named according to their absorption maxima at xxx nm.

CpcS1 is a relatively simple system. It is active as a monomer, and does not require cofactors. A more complex situation has been found with S-type lyases from other organisms classified as group I, where the concerted action of two subunits (CpcS and CpcU) is required (Saunée *et al*., 2008; Shen *et al*., 2008). CpcS1 from *Nostoc* binds the chromophore rapidly (∼0.1 s) and reversibly, and then transfers it in a much slower, irreversible reaction to the apoproteins (∼15 min) (Tu *et al*., 2008) (Fig. 3A). The optical properties of the CpcS1-PCB adduct are intermediate between free bilins and native phycocyanins: it fluoresces weakly, and the absorption is increased only moderately (i.e. about twofold). PCB is boundrelatively weakly, as might be expected for an intermediate: it is retained during Ni^2+^-affinity chromatography, but mostly lost during SDS-PAGE, tryptic digestion or mass-spectral analysis. It is unclear, therefore, if a weak, covalent bond or a relatively stable non-covalent bond is formed during the catalytic cycle. In the latter case, the small amounts of covalently bound PCB seen on SDS-PAGE would constitute a side product. The strongest evidence so far for a genuine covalent bond comes from the spectrophotometric absorption of the urea-denatured intermediate: it matches that of denatured PC and is at much shorter wavelengths than that of free PCB. CpcS1 also catalyses the addition of small molecules to PCB (J.M. Tu, S. Böhm, K.H. Zhao, H. Scheer, unpublished). Adducts with thiols and imidazole were isolated after incubation with PCB and CpcS1, and mercaptoethanol is also able to cleave PCB from the CpcS1 adduct. Thiols can add spontaneously to the 3-ethylidene group (Köst *et al*., 1975) or, at much higher thiol concentrations, to the central methine bridge of PCB (Kufer and Scheer, 1982). To our knowledge, spontaneous formation of an imidazole adduct has not been reported, but its formation by the lyase is intriguing, because conserved histidines have been found in several lyases (Zhao *et al*., 2005; 2006). Furthermore, a histidine is frequently found next to the binding cysteine of plant-type phytochromes, and even conserved in several bacterial phytochromes with N-terminal binding site (Wu and Lagarias, 2000; Lamparter, 2004). Chromophorylation by CpcS1 might then involve a histidine-bound intermediate. Because, as pointed out to us by L. Moroder (Martinsried), imidiazolides are relatively labile and can be cleaved by thiols (Shaltiel, 1967), this could be a model for the reaction catalysed by CpcS1.

The reaction catalysed by the E/F-type lyases is more complex (Fig. 3B). The E- and F-subunits form a 1:1 complex, which interacts not only with the α-subunit apoproteins, CpcA or PecA, but also with the chromophorylated proteins (Fairchild and Glazer, 1994b; Böhm *et al*., 2007). Both lyase subunits are required for chromophore attachment. The reaction has been studied in some detail with the isomerizing lyase, PecE/F from *Nostoc* PCC 7120. The F-subunit alone is inactive; the E-subunit alone catalyses only the PCB attachment without isomerization. Both subunits can bind PCB covalently, but the binding is even weaker than with CpcS1 (Zhao *et al*., 2005b), and it is again not yet clear if the lyase adducts are part of the catalytic cycle. PCB addition to the apoprotein by the E-subunit is slow, but is accelerated if a complex of the protein substrate with the E-subunit is formed prior to chromophore addition, indicating that protein–protein interactions are rate-limiting. These studies (Böhm *et al*., 2007) with the E-subunit also provided the first experimental evidence for a chaperone-like function that had previously been discussed for lyases (Schluchter and Glazer, 1999). PecE can transform a low absorption, low fluorescence spontaneous addition product, P645, to a product P641 that has the high absorption and high fluorescence typical for native biliproteins: P641 is the same product that is formed from PecA and PCB in the presence of the E-subunit. The F-subunit carries the isomerase activity, and a motif has been identified (Zhao *et al*., 2005b) that is also present in some lyases from marine picocyanobacteria and might therefore be related to PUB formation (Six *et al*., 2005) (see above). Interestingly, it contains a neighbouring histidine–cysteine pair that is essential for function and reminiscent of the lyase-binding-site motif of plant phytochromes (Wu and Lagarias, 2000). As the motif is absent in non-isomerizing EF-type lyases, this raises the question: what is the function of the F-subunit in non-isomerizing lyases? A further aspect is the inhibiting influence of Triton X-100 on the reaction catalysed by the isomerizing lyase. In the absence of the detergent, a mixture of the PCB-adduct and the PVB-bearing product, α-PEC, is formed but when present, Triton X-100 suppresses formation of the PCB-adduct (Zhao *et al*., 2002).

Another largely unaddressed issue is the bilin stereoselectivity of lyases. PCB isolated from PC by thermal treatment is mainly 3E-configured, while the 3Z-isomer is produced in cell extracts of *Cyanidium caldarium* (Beale and Cornejo, 1991). 3E-PCB binding kinetics under catalysis of an E/F-type are thought to involve, as rate-limiting step, the isomerization of the 3E- to the 3Z-isomer, the latter being the substrate for the fast binding reaction (Beale, 1993). The requirement of a thiol for catalysis by E/F-type lyases would be compatible with this idea, but experimental proof is still lacking.

## Chromophore detachment

Considerable amounts of biliproteins are degraded during conditions of reduced P, S or N supply, but surprisingly few mechanistic details are known (Grossman *et al*., 2003). Biliprotein degradation studies might benefit from the advances in biliprotein lyase research. Two proteins involved in the first stages of PBS degradation are NblA (Bienert *et al*., 2006; Zabulon *et al*., 2007) and NblB (Dolganov and Grossman, 1999), or related proteins identified by the non-bleaching phenotypes of the corresponding knockout mutants (Balabas *et al*., 2003). NblB is homologous to CpcE, sharing the HEAT-like motif^3^ but, to our knowledge, does not have chromophore-attaching activity.

NblA, which forms a dimer with four-helix bundle structure, seems to be directly involved in PBS degradation (Bienert *et al*., 2006). It specifically binds to α-CPC and α-PEC, which are considered crucial to maintain the trimeric, and possibly higher order, biliprotein structures. The interaction motif for complex formation has been mapped to the globin part of the biliprotein subunit, near ring D of the chromophore. The putative interaction site of the lyase, CpcE/F, is located on the same biliprotein face as the NbIA interaction motif, but in a different region near ring A of the chromophore (Zhao *et al*., 2007a). This raises the possibility that CpcE/F, which forms the thioether bond reversibly, also has a function in biliprotein degradation.

A complication with the non-bleaching phenotype arises from the high absorption of native as compared with denatured (or proteolytically degraded) biliproteins (see above). Therefore, bleaching is not necessarily related to loss of chromophores, but can also arise from proteolysis. Protease activity has neither been found for NblA nor for NblB, but other protease(s) may be involved. *Spirulina maxima* has, for example, a novel protease that could be responsible for the selective proteolysis of phycobiliproteins. It hydrolyses native CPC both in crude extracts and purified reconstructed systems (Nanni *et al*., 2001). It remains to be seen if such lyases are involved *in vivo* in biliprotein degradation, and how they relate to the proteases believed to degrade unmodified or incompletely matured biliproteins (see above).

## Phylogenetic relationships

Both subunits of the E/F-type lyases are α-helical proteins characterized by HEAT-like repeats^3^ that are distantly related to proteins like α-karyopherin with an armadillo structure (Zhao *et al*., 2005). A largely α-helical protein with this motif has been crystallized, but its function is unknown (Julien *et al*., 2006). The E- and F-subunits probably evolved from a single gene, but they have lower homologies to each other than orthologues of the individual subunits. Several marine picocyanobacteria contain genes coding for an E/F fusion protein (Six *et al*., 2005), linked by a conserved ‘adapter’ sequence. Biochemical proof for a lyase function of these chimeric proteins is still lacking. E/F-type lyases are also homologous to NblB, the protein previously discussed in relation to PBS degradation (Dolganov and Grossman, 1999).

Secondary structure prediction for S/U type lyases indicates the presence of α-helix and extended β-sheet in a 2:1 ratio, but also large amounts of random coil structure (H. Scheer and K.H. Zhao, unpublished). Homologies to proteins of known function and structure are very low; therefore, structure modeling is not possible, and X-ray structures are required. A phylogenetic analysis has recently been carried out with the S/U-type lyases (Shen *et al*., 2008). The S- and U-proteins are members of the same protein family. Two groups have been defined (CpeS and CpeU) that relate to PE synthesis, and four groups (CpcS-I, CpcS-II, CpcSIII, CpcU) that relate to PC synthesis. CpcS-I and CpcU occur always together in one clade of cyanobacteria, whereas CpcS-III belongs to another clade lacking a corresponding *cpcU* gene. This classification is supported by biochemical evidence: the monomeric, single-subunit CpcS1 from *Nostoc* PCC7120 (Zhao *et al*., 2007a) is a member of group III, while the group I CpcS from *Synechococcus* PCC7002 is inactive by itself, and active only in the form of a heterodimer with CpcU (Saunée *et al*., 2008; Shen *et al*., 2008). No biochemical studies are published with CpcS of clade II cyanobacteria.

There is no comparably detailed phylogenetic analysis for the T-type lyases. The homology to the S/U-type lyases is low, indicating a different origin, and there are again no homologous proteins of known function. Secondary structure predictions indicate again α-helix and extended β-sheet in a 2:1 ratio, but the amount of random coil is even higher that with the S/U-type lyases (H. Scheer and K.H. Zhao, unpublished).

Inactive members of the S/U and T lyase protein families with yet unknown functions have been found in two organisms (Zhao *et al*., 2007; Shen *et al*., 2008). It remains to be seen if such proteins form a defined subclass, and what their functions are. It is also still an open question how the large numbers of genes annotated as S- and T-type lyases present, for example, in *Gloeobacter* (Nakamura *et al*., 2003) relate to the biliproteins present (Bryant *et al*., 1981).

## Applications

The most obvious ‘application’ of lyase-based reconstitution systems is studies on biliprotein function. The lyases, for the first time, make it possible to modify the chromophore and protein separately, then linking them covalently by use of the appropriate lyase(s). Another emerging application in basic research is studies on protein folding, where covalently bound chromophores in a native system are desirable to avoid artefacts. Absorption, fluorescence and circular dichroism of biliproteins are modulated drastically during folding, and controlled by different properties of the protein. In particular, fluorescence has been proposed as an indicator for the protein dynamics, and absorption and Vis-CD as indicators for the tertiary structure (Kupka and Scheer, 2008). Subunits with different chromophores are attractive models to monitor folding by fluorescence resonance energy transfer. The β-subunit of R-PC is a natural prototype system for such studies, because it has two different chromophores (PCB, PUB) that are spectrally well separated (Ma *et al*., 2007). Lyases should give access to biliproteins with other chromophore pairs. Furthermore, combinations with PVB enable the chromophore to be switched between two states with different absorption and vastly different fluorescence characteristics: the 5Z-isomer is highly fluorescent, the 5E-isomer is practically non-fluorescent (Zhao *et al*., 1995).

An established practical application of biliproteins is their use as fluorescence labels. Classical applications of this kind require the use of holoproteins (Oi *et al*., 1982; Glazer and Stryer, 1983). The complete assembly has become feasible now, at least in *E. coli*, and might be transferable to other organisms of interest. It requires the multiplasmid-based production of: (i) the apoprotein, (ii) a heme-oxygenase, (iii) one or more reductases required to generate the chromophore from heme and (iv) the lyase(s) (Tooley *et al*., 2001; Tooley and Glazer, 2002; Zhao *et al*., 2007; Saunée *et al*., 2008). As an example, cyanobacterial genes encoding heme oxygenase (*ho1*), PCB:ferredoxin oxidoreductase (*pcyA*), the apoprotein (CpcA), and the two subunits of the heterodimeric lyase (CpcE/F) were produced in *E. coli* carrying two plasmids with the genes under control of the *trc* promoter. Upon induction, the cellular heme pool is used as a chromophore precursor to generate holo-α-CPC. A minimum system would then require introduction of four genes: a heme oxygenase, a reductase, a lyase (monomeric, single subunit S-type) and the apoprotein. For certain applications of this kind, the lyase might even be dispensed with and replaced by spontaneous chromophore addition (Hu *et al*., 2006), but the fluorescence yields are usually much better in the presence of a lyase. Even this is already considerably more complex than the green fluorescing protein and related systems, but biliproteins cover a broader spectral range, and have more intense absorption and fluorescence, which may compensate these disadvantages.

## Conclusions

The current state of knowledge on biliprotein lyases depicted in Figs 1 and 3 is still incomplete, and little is known of their regulation. However, the rapid progress after discovering the isomerizing activities for E/F-type lyases and lyase activities of the S/T/U-type gene products has reinvigorated research in the subject. Currently, work has mainly concentrated on cyanobacterial phyco(erythro)cyanins and allophycocyanins. However, methodological advances in lyase research, as well as the discovery of large numbers of homologues, in particular in PE-producing cyanobacteria, raise expectations that the relevant lyases for other biliproteins will soon be identified. This should open new research possibilities on the transfer from *in vitro* or *E. coli* studies to the cyanobacteria proper; on the subsequent assembly/reassembly of the PBS in cyanobacteria and red algae; on the assembly and organization of the cryptophyte light-harvesting system; and on applications in basic research like protein folding, and also in the use of phycobiliproteins for fluorescence labelling. It is likely that progress in lyases will also reinvigorate interest in biliprotein degradation.

## Footnotes

1 This modification has not been studied in detail, but can, for example, be inferred from a comparison of X-ray structures and gene sequences; compare pdb structure 1KN1 (Liu *et al*., 2005) and SWISSPROT entry P59856 (Kunimoto *et al*., 2003).

2 Consensus sequence, the actual position of the central conserved Cys varies from positions 81 to 84.

3 HEAT-like repeats (Pfam entry HEAT_PBS, PF03130) are members of the armadillo repeat superfamiliy (ARM, CL0019). They are short helical segments that arrange zig-zag in anti-parallel fashion, and are generally involved in protein–protein interactions.
